# Predicting Structural Susceptibility of Proteins to Proteolytic Processing

**DOI:** 10.3390/ijms241310761

**Published:** 2023-06-28

**Authors:** Evgenii V. Matveev, Vyacheslav V. Safronov, Gennady V. Ponomarev, Marat D. Kazanov

**Affiliations:** 1 Skolkovo Institute of Science and Technology, Moscow 121205, Russia; 2 A.A. Kharkevich Institute for Information Transmission Problems, Moscow 127051, Russia; 3 Dmitry Rogachev National Medical Research Center of Pediatric Hematology, Oncology and Immunology, Moscow 117998, Russia; 4 Faculty of Bioengineering and Bioinformatics, Lomonosov Moscow State University, Moscow 119991, Russia; 5 Faculty of Engineering and Natural Sciences, Sabanci University, Istanbul 34956, Turkey

**Keywords:** regulatory proteolysis, proteases, protease substrates, substrate identification

## Abstract

The importance of 3D protein structure in proteolytic processing is well known. However, despite the plethora of existing methods for predicting proteolytic sites, only a few of them utilize the structural features of potential substrates as predictors. Moreover, to our knowledge, there is currently no method available for predicting the structural susceptibility of protein regions to proteolysis. We developed such a method using data from CutDB, a database that contains experimentally verified proteolytic events. For prediction, we utilized structural features that have been shown to influence proteolysis in earlier studies, such as solvent accessibility, secondary structure, and temperature factor. Additionally, we introduced new structural features, including length of protruded loops and flexibility of protein termini. To maximize the prediction quality of the method, we carefully curated the training set, selected an appropriate machine learning method, and sampled negative examples to determine the optimal positive-to-negative class size ratio. We demonstrated that combining our method with models of protease primary specificity can outperform existing bioinformatics methods for the prediction of proteolytic sites. We also discussed the possibility of utilizing this method for bioinformatics prediction of other post-translational modifications.

## 1. Introduction

After synthesis, proteins within living cells undergo a wide range of chemical modifications collectively referred to as post-translational modifications (PTMs) [1]. To date, about a dozen types of PTMs are known, including phosphorylation, acetylation, glycosylation, ubiquitination, methylation, and others [2]. Unlike most types of post-translational modifications, which involve the addition of small chemical groups, proteolysis is an irreversible post-translational modification that catalyzes the hydrolysis of the peptide bond [3]. To perform cleavage, a protease needs to bind to the polypeptide chain in the vicinity of the cleaved peptide bond [4]. The ability of a protease to bind to a polypeptide chain in a specific amino acid context is known as protease specificity, which is an intrinsic property of the protease’s active site pocket [5]. The broad or narrow specificity of a protease refers to its ability to cleave a wide or restricted range of substrate sequences, respectively. As proteases have evolved to fulfill specific biological functions, the variations in protease specificity can be attributed to evolutionary adaptation [6]. Thus, proteases with broad specificity usually participate in processes such as protein degradation or processing, breaking down proteins into smaller peptides and amino acids. On the other hand, regulatory proteases typically possess narrow specificity, allowing precise cleavages of specific proteins in specific cellular contexts to regulate signaling pathways or protein activation/inactivation [7]. The 3D structure of substrates is not crucial for digestive proteases since the substrate is cleaved into short peptides, losing its native 3D structure. However, it plays a significant role in regulatory proteolysis, as potential cleavage sites can be shielded within the hydrophobic core of the protein [8,9,10].

Our understanding of regulatory proteolytic networks in multicellular organisms is still limited, making it crucial to conduct further research in this direction. Experimental work to identify protease substrates and cleavage sites is highly labor-intensive. However, the guidance provided by bioinformatics predictions can significantly facilitate this process. To date, numerous bioinformatics methods exist for predicting proteolytic cleavage sites [11,12,13,14,15,16,17,18,19,20]. However, many of these methods primarily focus on modeling protease sequence preferences near the cleavage site and often do not incorporate information about the 3D structures of potential substrates [21]. While some methods incorporate specific structural features of the substrate into their prediction models [17,22], to our knowledge, only one method considers the 3D structure of the substrate as an input [23]. However, even this method does not provide separate probabilities for the structural susceptibility of the peptide bonds of the considered protein to proteolysis. The Nickpred method [8], which was originally developed for this purpose, is no longer available. To address this gap in the field, we developed a method for predicting the susceptibility of protein regions to proteolysis based on the known 3D structure of the potential substrate.

## 2. Results

### 2.1. The Three-Dimensional Structure of a Protease Substrate Determines the Susceptibility of Protein Regions to Proteolysis

**Figure 1:**
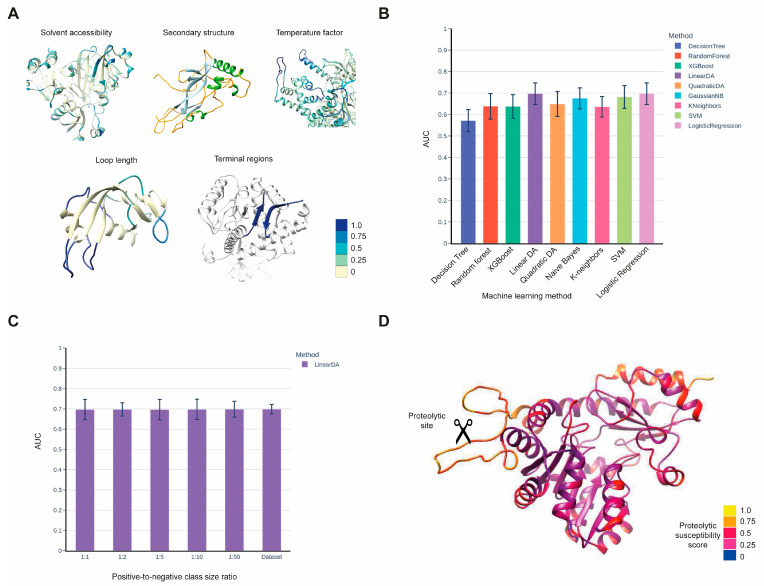
(**A**) List of structural features used in the method, along with examples of their distribution along the protein polypeptide chain visualized within the substrate structures. The color bar represents a color scale ranging from 0 to 1, indicating numerical features such as solvent accessibility, temperature factor, and loop length, as well as binary features such as terminal regions. The secondary structure is visualized using a different color scheme: helices are shown in green, beta strands in light blue, and loops in yellow. (**B**) Prediction quality, measured using the Area Under the ROC Curve (AUC), of various machine learning methods calculated via cross-validation using the training set of CutDB proteolytic events mapped onto PDB structures. Negative class examples were sampled to achieve a 1:1 positive-to-negative class size ratio. (**C**) Dependence of the method’s prediction quality on different positive-to-negative class ratios. (**D**) Visualization of the proteolytic susceptibility probabilities predicted by our method for the 3D structure of the protease substrate.

Our aim was to develop a method that predicts the susceptibility of protein regions to proteolysis. This method takes the three-dimensional structure of a potential protease substrate as input and provides a cleavage susceptibility score for each peptide bond of the protein. Our predictive model was constructed using experimentally verified proteolytic events extracted from the CutDB [24]. We mapped the proteolytic sites from CutDB onto the available 3D structures of substrates extracted from the Protein Data Bank (PDB) [25] to create a training set. For each peptide bond, we calculated the structural features that were identified in earlier studies as predictors of proteolytic susceptibility [26,27]. The set of structural features used for prediction included well-known predictors such as solvent accessibility, secondary structure, and B-factor, as well as additional features developed by our group, including loop length and regions of flexible N- and C-termini (see Figure 1 and Methods for a complete list of features). We estimated the prediction quality of the developed method using the cross-validation technique on the training set and a separate testing dataset, which was collected from recent literature on proteolytic cleavage experiments. To maximize the prediction quality, we applied eight machine learning methods and ultimately selected Linear Discriminant Analysis (Figure 1B). Our training set was highly imbalanced, consisting of 445 positive examples (cleavage sites) and 68,840 negative examples. Therefore, we sampled the negative class in various proportions relative to the size of the positive class and examined the impact of the positive-to-negative class size ratio on the prediction quality (Figure 1C and Figure S1). We found that the quality of the prediction was generally independent of the class size ratio; thus, to simplify calculations, we chose a 1:1 class size ratio (Figure 1C). We also visualized the predicted cleavage susceptibility scores on the 3D structures of substrates and confirmed that the method assigns higher scores to protruded loops, regions with a high B-factor, N- and C- flexible termini, and solvent-accessible regions (Figure 1D), as expected from earlier studies [26,27].

### 2.2. Extension of the Training Set with AlphFold Models Improves the Quality of Prediction

**Figure 2:**
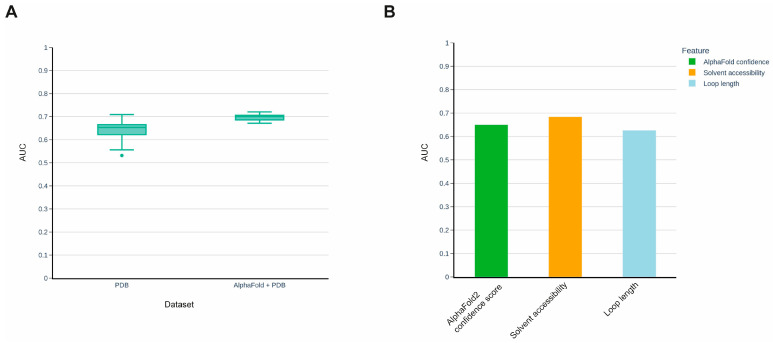
(**A**) Improvement in prediction quality of the method after extension of the training set using AlphaFold models. (**B**) Comparison of AlphaFold confidence score with solvent accessibility and loop length in predicting proteolytic sites.

Recent progress in artificial intelligence has led to breakthroughs in various fields of study, including computational molecular biology. Thus, the recently introduced protein structure prediction method AlphaFold has significantly outperformed other methods in this field and has demonstrated a prediction quality comparable to experimental methods [28]. Later, AlphaFold was applied to the entire human proteome, and high-quality predicted 3D structures were made publicly available in the AlphaFold Protein Structure Database [29]. We used 3D protein structures predicted by AlphaFold to expand our training set and construct a new model with the aim of comparing its prediction quality to the previous version of the model, which was solely based on PDB 3D structures. However, not all structural features extracted from PDB 3D structures are available in AlphaFold models, notably, experimental-specific features such as the temperature factor (B-factor). Therefore, we reconstructed our initial PDB-based model, excluding the experiment-specific features, and compared its performance with the model constructed using the training set extended with AlphaFold-predicted 3D structures (Figure 2A). The latter model demonstrated better prediction quality and the difference between the median values of the models’ Area Under the Curve (AUC) of the Receiver Operating Characteristic curve (ROC) [30] was 0.05. It is worth noting that the AlphaFold method provides values for the prediction confidence for each amino acid position of the protein. We analyzed whether this feature alone could predict susceptibility to proteolysis and found that it possesses substantial predictive power (Figure 2B).

### 2.3. Comparison to Other Proteolytic Site Prediction Methods

**Figure 3:**
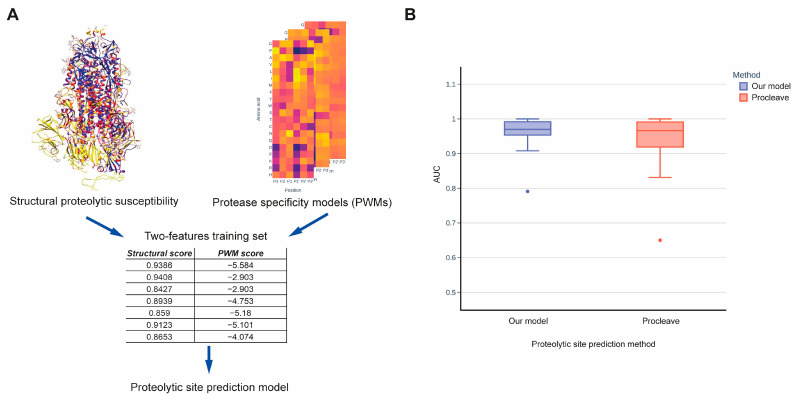
(**A**) A schematic representation of combining the proteolytic susceptibility probabilities predicted by our 3D structure-based method with protease sequence specificity models for comparison with other proteolytic site prediction methods. (**B**) Comparison of prediction quality between our method combined with protease sequence specificity models and the Procleave method.

To the best of our knowledge, our method is the first to estimate the structural susceptibilities of protein regions to proteolysis regardless of specific proteases. Thus, there is currently no method available to directly compare prediction qualities. However, if we add knowledge on protease specificity into our method, for example by using a position-specific scoring matrix (PSSM) [31,32], we can compare our method with 3D structure-based methods that also incorporate information on protease specificity. To this end, we chose Procleave [23], the most recent and reliable method for the identification of proteolytic sites, for comparison. This method can predict cleavage sites for 27 proteases, including matrix metalloproteases, cathepsins, and other proteinases. We generated PSSM matrices for these proteases using data from the MEROPS database (see Section 4 [33]. To integrate the structural susceptibility predicted by our method with protease sequence specificity, we created a dataset that included two features: the structural score and the PSSM score. This dataset was used to train the prediction model using data from CutDB (Figure 3A). The obtained model demonstrated improved performance on the testing set compared to the Procleave method (Figure 3B). The AUC ROC mean values were 0.962 and 0.937, respectively, while the respective median values were 0.97 and 0.966. However, the statistical difference estimated using the Wilcoxon test was not found to be significant.

## 3. Discussion

In this study, we presented a method for estimating structural susceptibility to proteolysis protein regions based on the known three-dimensional structure of a protein. It is known that the 3D structure of the protease substrate significantly influences the ability of protease to cleave a protein’s peptide bonds [8,9,10,26,34,35]. Indeed, it is a common opinion that protein regions in the hydrophobic core of a protein are hardly accessible to proteolytic processing while the 3D structure of the protein is intact [8,9,10]. Another protein property that influences proteolytic processing is the secondary structure: our [26,27] and other [8,9,10,34,35] studies showed that loops are cleaved more easily than helices, and helices are cleaved more easily than beta-sheets. These and other known structural preferences of limited proteolysis seem universal for different types of proteases, contrary to protease sequence specificity [26]. Although several proteolysis prediction tools use specific structural features, there is, to our knowledge, no method that estimates the general susceptibility to proteolysis of protein regions based on known 3D structure. We developed and presented here such a type of method to fill this gap in the field.

We incorporated into the method structural features that influenced proteolytic processing according to current knowledge from our [26,27] and previous [8,9,10,34,35] studies. We also developed two additional structural features—loop length and N- and C- termini regions—based on our earlier observations [26]. To maximize prediction quality, we tried several machine learning methods and chose Linear Discriminant Analysis, which showed the best results for our task. Together, these efforts allowed us to develop a method demonstrating a quality of prediction comparable with state-of-the-art proteolytic site prediction tools, such as Procleave, when combined with protease primary specificity models.

Our method estimates the susceptibility of being proteolytically processed for each peptide bond of the considered protein with a known 3D structure. As the number of cleavages performed in a protein by a particular protease depends on its colocalization and the colocalization duration [36,37], we did not apply any threshold to the predicted cleavage probability. Thus, our method did not classify protein peptide bonds into presumably cleaved and uncleaved ones. Moreover, after the first cleavage, a substrate can change its conformation or even become denatured; thus, predicted proteolytic sites can lose their confidence [38]. A new round of prediction is preferable if the 3D structure of the protein after conformations induced by the first cleavage changes is known.

Future research in proteolytic site prediction could focus on developing a method that combines prediction of structural susceptibility of protein regions, using the methodology developed in this study as a universal component applicable to all proteases, along with protease-specific models as plug-in modules. In this study, we demonstrated the relevance of this approach. In conclusion, we speculate that the scope of our method extends beyond the prediction of proteolytic events to encompass other post-translational modifications. Indeed, our method assigns higher probabilities of proteolytic sites to the hydrophilic exterior rather than the hydrophobic core of the protein, to protruded loops rather than helices and beta-sheets, and to flexible protein regions instead of the stable parts of the protein structure. This trend may hold true for various other post-translational modifications.

## 4. Materials and Methods

### 4.1. Data Collection and Processing

Information on experimentally verified proteolytic events was extracted from CutDB [39]. For each proteolytic event, we considered three attributes: the substrate identifier, the position of the proteolytic site within the substrate sequence, and the protease MEROPS code (Supplemental File S1). In total, we extracted 4576 proteolytic events related to 2062 unique substrates cleaved by 457 proteases. All substrate sequences were collected into a single FASTA file and then queried against the PDB database [25] using BLAST [40]. If the retrieved results included structures with a sequence identity of over 90% of the queried substrate sequence, the top structure in the list was associated with the protease substrate. Otherwise, the substrate was categorized as unmapped. In total, we found 585 three-dimensional structures of substrates associated with 1499 proteolytic events cleaved by 256 proteases. To map substrate amino acid positions into the 3D structure, we aligned substrate and 3D structure sequences using Clustal Omega [41]. The number of proteolytic sites upon mapping decreased by more than twofold (777 proteolytic sites, 323 structures, 183 proteases), as many of them were mapped into disordered regions. Since some of the secondary proteolytic cleavages observed in the experiments could occur after the loss of the intact substrate’s 3D structure, we visualized the cleavage sites on the 3D structures using Chimera [42] and performed manual curation. We excluded proteolytic events if there were multiple cleavages attributed to a single publication and if they were predominantly located within the hydrophobic core of the substrate, indicating a potential loss of the 3D structure during the experiment. The final training dataset comprised 445 proteolytic events, specifically associated with peptide bonds in 190 3D structures of substrates that underwent proteolytic processing by 130 proteases. The average number of proteolytic events per protein in the training set was 2.34, with a median of one.

### 4.2. Structural Features

The selection of structural features for use in the prediction model was based on our earlier studies [26,27] as well as other relevant research in the field [8,9,10,34,35]. Primary structural features were solvent accessibility, secondary structure, and temperature factor (B-factor). Solvent accessibility and secondary structure were obtained using the DSSP tool [43]. B-factor was extracted from the PDB files of protease substrates’ 3D structures. Based on our earlier observations of an increased density of cleavage sites in long protruded loops and C- and N-protein termini, we introduced two specific structural features associated with these observations. First, we added the length of the loop as a feature and assigned it to all peptide bonds within the loop. Second, we defined the regions of the C- and N-protein termini as unstructured terminal protein regions that are adjacent to the regular secondary structure elements, with the exception of short ones (see comments in the source code). Solvent accessibility, loop length, and B-factor were normalized using min-max scaling. The secondary structure was converted into three binary features that indicated the presence or absence of specific types of secondary structures. C- and N-protein termini were encoded as a binary variable. A single binary variable was used to represent the C- and N-protein termini.

### 4.3. Training Set Processing and Selection of the Machine Learning Method

To create the training set, we computed the mentioned structural features for every amino acid in each substrate. Next, we assigned the structural features calculated for the amino acid at P1 position of the cleavage sites (Schechter–Berger notation) [44] to each peptide bond, as this position has previously been identified as the most important from a structural perspective [26,45,46,47]. We applied multiple machine learning methods from the scikit-learn library [48], such as Random Forest, Decision Trees, Naïve Bayes, SVM, Logistic Regression, XGBoost, Linear and Quadratic Discriminant Analysis, to identify the optimal method for our task (Figure 1B). The quality of the models was assessed via the AUC ROC metric using a 10-fold cross-validation technique. We also varied the proportions of the negative class relative to the positive class to assess the impact of the positive–negative class size ratio on the prediction quality and determine the optimal ratio (Figure 1C). We found that the quality of prediction was generally independent of the class size ratio; therefore, we chose a 1:1 class size ratio. Among the applied machine learning methods, Linear Discriminant Analysis demonstrated the best quality of prediction.

### 4.4. Extending Training Set with AlphaFold Models

Structure models were downloaded from the AlphaFold Protein Structure Database [29]. BLAST [40] was used to query the remaining protease substrates against the AlphaFold models. Filtering of the BLAST search results, mapping of the cleavage sites into AlphaFold models, and visualization followed a similar procedure as described above for the search against PDB. The numbers of proteolytic sites, substrates, and associated proteases at each filtering step were as follows: 3168, 1209, and 317 after the BLAST search step; 2925, 1209, and 317 after the mapping step; and 2918, 1205, and 314 after the curation step, respectively. In this dataset, proteins had an average of 2.42 proteolytic events, with a median of one event per protein.

### 4.5. Combining the Method with Protease Sequence Specificity Models

A testing set of proteolytic events was created using the MEROPS database [33]. We selected proteolytic events that were added to the database after the release of the Procleave method [23]. At each filtering step, the counts of proteolytic sites, substrates, and associated proteases were as follows: 213, 129, and 3 after extraction from MEROPS; 81, 48, and 3 after BLAST search; 43, 27, and 3 after mapping; and 28, 18, and 3 after the curation process, respectively. In the testing set, the average number of proteolytic events per protein was 2.34, while the median value was one. Protease sequence specificity models, in the form of PSSM matrices [31], were constructed following the method described in [49]. To combine the predicted values of structural susceptibility to proteolysis generated by our method with the sequence specificity scores generated by PSSM models, we created a training set that included these two features and applied the Naïve Bayes method. The obtained model was applied to the testing set and compared with the Procleave results.

## Data Availability

Data and source code are available at: https://github.com/KazanovLab/ProteolysisStructuralPrediction (accessed on 15 June 2023). Development scripts are available at: https://github.com/EugeneVlg02/ProteolysisStructuralPrediction_development (accessed on 15 June 2023).

## Supplementary Materials

The supporting information can be downloaded at: https://www.mdpi.com/article/10.3390/ijms241310761/s1.
