# Supplementary material for: Predicting Structural Susceptibility of Proteins to Proteolytic Processing
Source: Int J Mol Sci. 2023 Jun 28;24(13):10761. doi: 10.3390/ijms241310761 (PMC10342023; doi:10.3390/ijms241310761)
Supplement: Supplementary file 1 [file ijms-24-10761-s001.zip › ijms-2448357-supplementary.pdf]

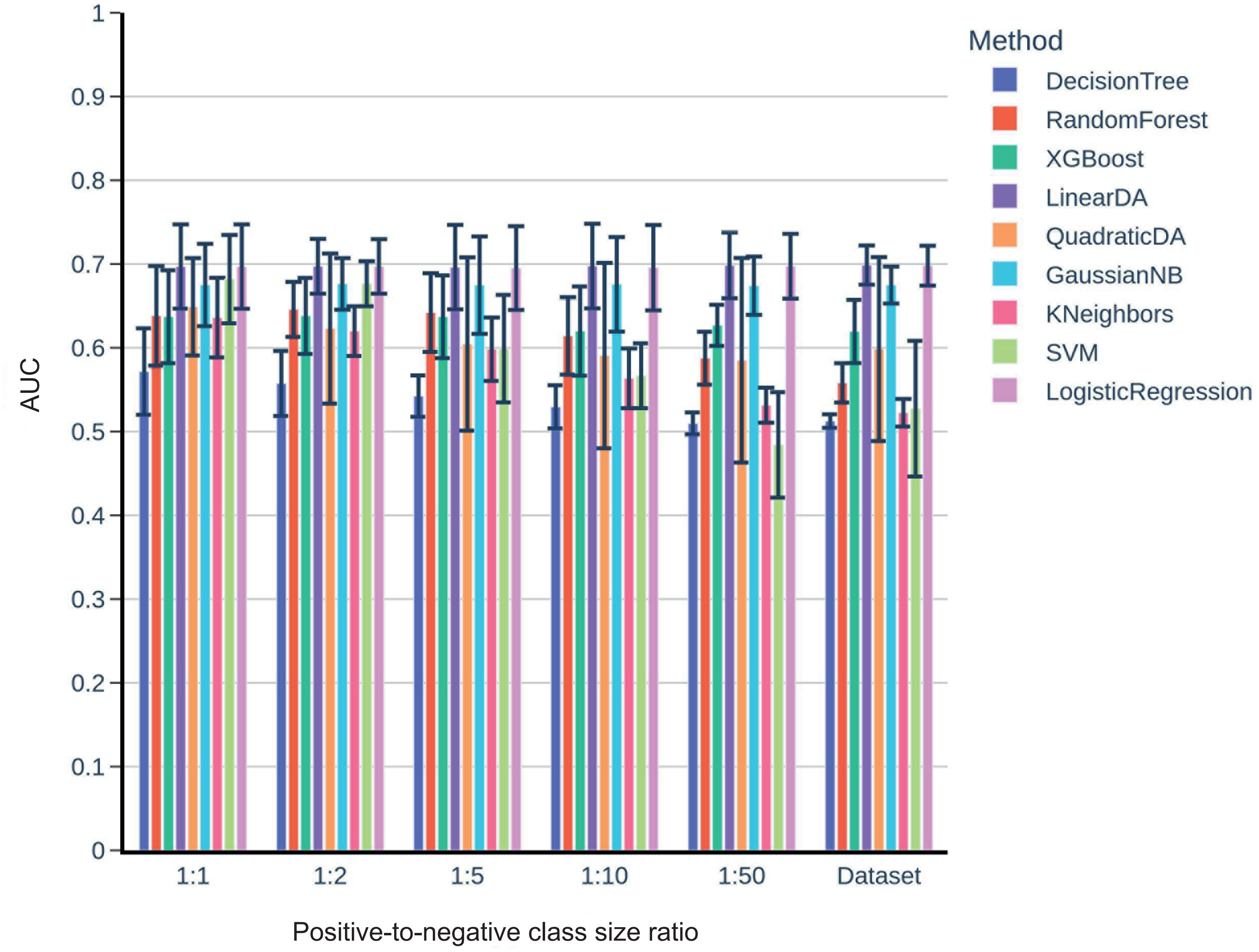

Figure S1. Dependence of the method’s prediction quality from the different positive-to-negative class ratios.
